# A major root-associated acid phosphatase in *Arabidopsis*, AtPAP10, is regulated by both local and systemic signals under phosphate starvation

**DOI:** 10.1093/jxb/eru377

**Published:** 2014-09-20

**Authors:** Ye Zhang, Xiaoyue Wang, Shan Lu, Dong Liu

**Affiliations:** MOE Key Laboratory of Bioinformatics, School of Life Sciences, Tsinghua University, Beijing 100084, China

**Keywords:** Phosphate starvation responses, purple acid phosphatase 10, local and systemic signalling, ethylene, sucrose, *Arabidopsis*.

## Abstract

The induction and secretion of acid phosphatases (APases) is a universal response of plants to phosphate (Pi) starvation. AtPAP10 (*Arabidopsis* purple acid phosphatase 10) is a major Pi starvation-induced APase that is associated with the root surface in *Arabidopsis*. So far, the roles of local and systemic signalling in regulating root-associated AtPAP10 activity remain largely unknown. In this work, we show that a decrease of local, external Pi availability is sufficient to induce *AtPAP10* transcription in roots in the presence of sucrose, a systemic signal from shoots, whereas the magnitude of the induction is affected by the Pi status of the whole plant. Once the *AtPAP10* mRNAs are synthesized in roots, subsequent accumulation of AtPAP10 proteins in root cells and increase in AtPAP10 activity on the root surface are mainly controlled by local signalling. Previously, ethylene has been demonstrated to be a positive regulator of AtPAP10 activity. In this study, we provide evidence that under Pi deficiency ethylene mainly modulates enzymatic activity of AtPAP10 on the root surface, but not *AtPAP10* transcription and protein accumulation, suggesting that it functions as a local signal. Furthermore, our work indicates that the effect of ethylene on the induction of root-associated AtPAP10 activity depends on sucrose, but that the effect of sucrose does not depend on ethylene. These results reveal new insights into the distinct roles of local and systemic signalling in the regulation of root-associated AtPAP10 activity under Pi starvation.

## Summary

Under phosphate starvation, the root-associated activity of *Arabidopsis* acid phosphatase AtPAP10 is differentially regulated by local and systemic signalling at transcriptional, post-transcriptional, and post-translational levels.

## Introduction

Similarly to other essential plant nutrients, phosphorus (P) plays a pivotal role in plant growth and development (Marschner, 1995). In most soils, however, the concentration of inorganic phosphate (Pi), the major form of P that plants uptake through roots, is far below the level required for optimal plant growth (Raghothama, 2000; Vance *et al.*, 2003). As a countermeasure, plants have evolved sophisticated adaptive responses to enhance Pi acquisition and utilization efficiency. These responses include changes in root architecture, enhanced Pi transporter activities on the root surface, induction and secretion of acid phosphatases and ribonucleases, release of organic acids into the rhizosphere, and accumulation of starch and anthocyanin (Lynch and Brown 2008; Shen *et al.*, 2011; Yuan and Liu, 2008).

Regulation of these adaptive responses involves a complex network of both local and systemic signalling. In the case of Pi responses, the term “local signalling” generally refers to a response that is controlled by local Pi status rather than by Pi status of a remote part of the plant or of the whole plant. Researchers have proposed that the remodelling of root growth under Pi deficiency is triggered by the decrease in the local Pi levels outside the root (Ticconi *et al.*, 2004; Thibaud *et al.*, 2010). In this locally induced remodelling process, ethylene probably plays an important role (Nagarajan and Smith, 2012). Indeed, the expression of some genes involved in ethylene biosynthesis and signalling are locally induced by Pi starvation (Thibaud *et al.*, 2010). Locally induced genes also include those transcription factors linked to development (Myb, MADS), stress responses (WRKY), and cell wall synthesis (Thibaud *et al.*, 2010). In contrast to the expression of locally induced genes, the expression of many other phosphate starvation-induced (PSI) genes in roots is affected by the Pi status in shoots or in the whole plant, i.e., they are controlled by so-called “systemic signalling”. These systemically regulated genes are mainly involved in Pi transport, Pi recycling, and Pi signalling (Liu *et al.*, 1998; Burleigh and Harrison, 1999; Franco-Zorrilla *et al.*, 2005; Hou *et al.*, 2005; Thibaud *et al.*, 2010). Sucrose and miRNA399 have been considered to be two systemic signals that are transported from shoot to root to regulate PSI responses in root (Liu *et al.*, 2005, 2010; Chiou and Lin, 2011; Lei *et al.*, 2011*a*).

One of the hallmark responses of plants to Pi starvation is the induction and secretion of acid phosphatases (APases) (Tran *et al.*, 2010*a*). The PSI extracellular or secreted APases are believed to release Pi groups from organophosphates in the external environment. In natural and agricultural systems, most P exists in the form of organophosphates that are derived from decomposed plants or microorganisms (Bieleski, 1973). Thus, the enhanced secretion of APases increases the availability of Pi for root absorption. Several PSI-secreted APases have been biochemically and molecularly characterized in vascular plants, including those in white lupin (*Lupinus albus;* Ozawa *et al.*, 1995; Li and Tadano, 1996; Miller *et al.*, 2001), tomato (*Solanum lycopersicum;* Bozzo *et al.*, 2002, 2006), common bean (*Phaseolus vulgaris;* Liang *et al.*, 2010), tobacco (*Nicotiana tabacum;* Lung *et al.*, 2008), and *Arabidopsis thaliana* (Veljanovski *et al.*, 2006; Tran *et al.*, 2010*b*; Wang *et al.*, 2011). Purple acid phosphatases (PAPs) represent a unique group of APases, which are pink or purple in water solution when purified. In *Arabidopsis*, there are 29 members of this APase family (AtPAP), and the expression of at least 11 members is transcriptionally up-regulated by Pi starvation (del Pozo *et al.*, 1999; Haran *et al.*, 2000; Li *et al.*, 2002; Wang *et al.*, 2011; Zhu *et al.*, 2005). Our previous work has shown that accumulation of both mRNAs and proteins of one member of the AtPAP family, AtPAP10, is enhanced by Pi starvation (Wang *et al.*, 2011). Under Pi starvation, AtPAP10 is predominantly retained on the root surface rather than being released into the growth medium. In *Arabidopsis*, AtPAP10 is a major root-associated APase induced by Pi starvation. The root surface-associated AtPAP10 activity is also enhanced by sucrose and ethylene (Lei *et al*., 2011*a*, 2011*b*). Analyses of *atpap10* mutant and overexpressing lines indicated that AtPAP10 plays an important role in utilization of external organophosphates (Wang *et al*., 2011, 2014; Wang and Liu, 2012).

Regulation of root-associated AtPAP10 activity by Pi starvation may occur at multiple levels, including transcription of *AtPAP10* mRNA, accumulation and secretion of AtPAP10 proteins, and modulation of AtPAP10 enzymatic activity. The roles of local and systemic signalling in regulating this hallmark Pi response, however, remain largely unknown. In this study, we investigated how local and systemic signalling participate in the key regulatory steps to control the induction of AtPAP10 activity under Pi starvation. We also determined the functional relationship between sucrose and ethylene in their regulation of AtPAP10 activity. Because AtPAP10 is a major root-associated APase and plays a prominent role in plant adaptation to Pi deprivation, a thorough understanding of how its activity is controlled by local and systemic signalling will help us to better understand the molecular mechanism that governs plant Pi responses.

## Materials and methods

### Plant materials and growth conditions

All *Arabidopsis* plants used in this study, including the mutants and transgenic plants, were in the Columbia ecotype background. The Pi-sufficient medium (P+) used in this study contained half-strength MS salts (Murashige and Skoog, 1962) with 1% (w/v) sucrose and 1.2% (w/v) agar (Sigma-Aldrich Co., St. Louis, MO, catalogue no. A1296). In the Pi-deficient medium (P–), the 1.25mM KH_2_PO_4_ in the P+ medium was replaced with 0.65mM K_2_SO_4_. Seeds were surface sterilized with 20% (v/v) bleach for 10min and washed with distilled water three times. The seeds were then sown on the Petri plates containing P+ or P– medium. After 2 d of stratification at 4 °C, plates were vertically placed in the growth room with a photoperiod of 16h of light and 8h of darkness at 22–24 °C. The light intensity was 100 µmol m^–2^ s^–1^.

For liquid culture of *Arabidopsis* seedlings, seeds were surface sterilized as described above and stratified at 4 °C for 2 d before they were placed in 500ml conical flasks containing sterile P+ or P– liquid medium. The flasks were placed on a horizontal rotator at 50rpm and with the same lighting and temperature conditions as for the growth of plants on agar plates.

### Split-root experiments

The primary roots of 5-day-old *Arabidopsis* seedlings were removed to induce the formation of lateral roots. After another 12 d, the seedlings were transferred to two-compartment plates such that one lateral root was placed on P+ medium in one compartment, and the other lateral root was placed on P– medium in the other compartment. As the negative and positive controls, we transferred seedlings to similarly compartmented plates with both lateral roots on P+ medium or P– medium.

### Generation of triple mutant of *atpap12/15/26*

The triple mutant of *atpap12/15/26* was generated through genetic cross using the T-DNA insertion lines of GK-151C09 (*atpap12*), SALK_061597 (*atpap15*), and SALK_152821 (*atpap26*).

### Analysis of root-associated APase activity

For histochemical staining of APase activity on the root surface of *Arabidopsis* seedlings, an agar solution (0.5%, w/v) containing 0.01% (w/v) BCIP was evenly overlaid on the roots grown on the agar plates (Lloyd *et al.*, 2001). After 12h of colour development, the roots were photographed with a camera attached to a stereomicroscope (Olympus SZ61).

Root-associated APase activity in roots was quantified according to Wang *et al.* (2011).

### Quantitative real-time PCR analysis

Quantitative real-time PCR analysis of *AtPAP10* gene expression was carried out as described by Wang *et al.* (2011). The amounts of PCR products in each sample were normalized using the *ACTIN* gene as the internal control. The primers used for amplification of *AtPAP10*, *AT4*, *IPS1*, and *ACTIN* are listed in Supplementary Table S1.

### Generation of monoclonal antibodies and western blot analysis

Monoclonal antibodies against AtPAP10 proteins were generated at Abmat Company (Shanghai, China) using a synthetic peptide with the sequence of PDHDNRRWDS. The high specificity of the antibodies was confirmed by analysing the AtPAP10 protein levels in the WT, two *atpap10* mutants (*nop1-1* and *nop1-2*), and an *AtPAP10* overexpressing line (the *nop1-1* line transformed with a *35S-AtPAP10* construct) (See Supplementary Fig. S1). Western blot analysis of AtPAP10 proteins was performed according to Wang *et al.* (2011).

### Quantitative analysis of cellular Pi content

The cellular Pi contents were determined using the method described by Ames (1966). Briefly, the pre-weighed fresh shoot and root tissues were submerged in 1ml of 1% glacial acetate and frozen/thawed eight times. One hundred microlitres of extract was mixed with 200 µl of water and 700 µl of Pi reaction buffer (A: 0.48% NH_4_MoO_4_, 2.85% (v/v) H_2_SO_4_, B: 10% (w/v) ascorbic acid, A:B (v/v)=6:1). The reaction was proceeded at 37 °C for 1h. The Pi content was determined at A_820_ according to a premade standard curve and was expressed as µmol g^–1^ fresh weight.

### Quantitative analysis of sucrose content

Quantification of sucrose content in root tissues was performed according to Luo *et al.* (1998) with some modifications. Basically, five-day-old seedlings grown on P+ medium were transferred to P– medium and were then grown in the dark. Eight days after transfer, the roots were excised and ground in liquid nitrogen. Soluble sugar was extracted twice in 80% (v/v) methanol at room temperature. The extracted samples were centrifuged at 13 500 *g* for 10min, and the supernatant was dried under a nitrogen gas flow. Sucrose in the samples was quantified with an Agilent 1290 liquid chromatographer (Agilent, USA) equipped with a triple-quadruple tandem mass spectrometer (Agilent 6460, USA). A UK-Amino column (150×4.6mm, 3 µm) was used for liquid chromatography separation. Sucrose purchased from Sigma (cat. no. 84097) was used as the standard for quantification.

### Method for statistical analysis

The two-sample *t*-test function and one-way ANOVA function of Origin software (Origin Lab Corporation, Northampton, USA) was used to perform statistical analysis of the data generated in this work.

## Results

### Induction of root-associated AtPAP10 activity is independent of Pi status of the whole plant

**Fig. 1.**
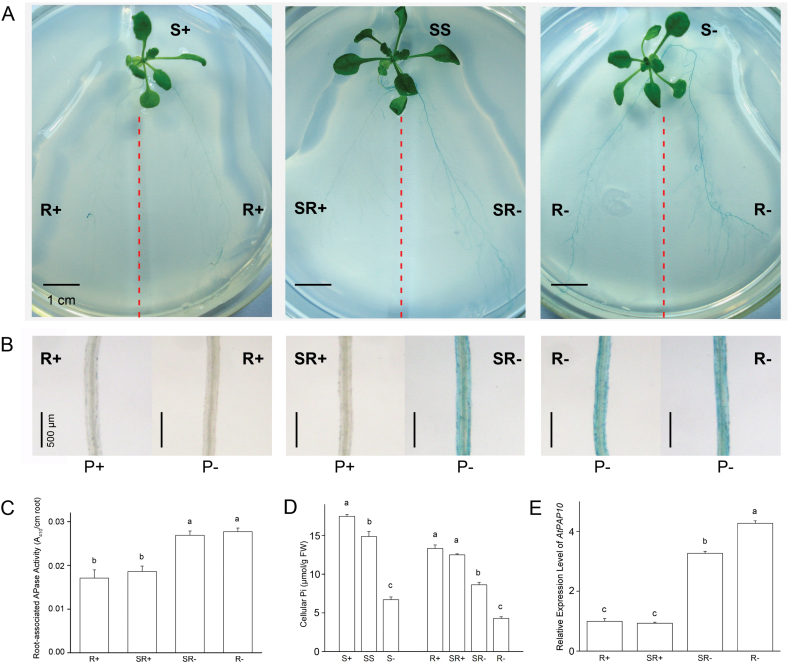
Root-associated AtPAP10 APase activity and transcription of *AtPAP10* gene in split-root assays. Seventeen-day-old seedlings were transferred to dishes with two compartments such that one half of each root system was placed in one compartment and the other half was placed in the other compartment. Four days after transfer, the roots were stained with BCIP. (A) APase activity on the surface of the split roots growing on P+ or P– medium in the compartmentalized dishes as indicated by BCIP staining. (B) Close views of the stained roots in A. (C) Root-associated APase activity of the *atpap12/15/26* triple mutant in the split-root experiment. (D) Cellular Pi contents of the shoots and roots of the seedlings in the split-root experiment. (E) Relative expression levels of *AtPAP10* mRNA in the roots of the seedlings in the split-root experiment. In C, D, and E; R+: Split roots in P+/P+ dishes; R–: Split roots in P–/P– dishes. SR+: Split roots on the P+ side of P+/P– dishes; SR–: Split roots on the P– side of P+/P– dishes; S+: Shoots grown on P+/P+ dishes; SS: Shoots grown on P+/P– dishes; S–: Shoots grown on P–/P– dishes. Values are the means±SEM of three replicates. Each replicate contained four split roots. A one-way ANOVA was carried out for the whole data set, and *post hoc* comparisons were conducted using the SPSS Tukey HSD test at *P*<0.05 level. Significant differences are indicated by different letters above the bars.

Pi starvation-induced AtPAP10 activity on the root surface (or root-associated AtPAP10 activity) can be specifically detected as blue precipitate by applying an agar solution containing 0.01% of APase substrate BCIP (5-bromo-4-chloro-3-indolyl-phosphate) to the root surface (Wang *et al*., 2011, 2014). To determine the roles of local and systemic signalling on the induction of root-associated AtPAP10 activity, we performed split-root experiments (see Materials and methods for detailed procedure of split-root experiments). The root grown on P+ medium in the compartmented agar plate was designated split root P+ or SR+ and that grown on P– medium was designated as split root P– or SR–. As the negative and positive controls, seedlings were grown on similar compartmented plates with both roots placed on P+ medium (designated R+) or P– medium (designated R–). Four days after growing on compartmented plate, no BCIP staining was evident on SR+ or R+, whereas a strong dark blue staining was evident on SR– and R– (Fig. 1A, B). The staining intensity of SR– was similar to that of R–.

We quantified the APase activity on the root surface for the samples collected from split-root experiments as shown in Fig. 1A. Because the generic substrate used for quantitative analysis, *p*NPP (*p*-nitrophenol phosphate), could also be catalysed by APases on the root surface other than by AtPAP10, we used a triple knockout mutant *atpap12/atpap15/atpap26* for this analysis. In this triple mutant, the total root-associated APase measured by *p*NPP was about 50% of that in the wild type (WT) under both P+ and P– conditions (Supplementary Fig. S2). Previously, we showed that AtPAP10 accounted for about 30% of total APase activity on the root surface as measured by *p*NPP (Wang *et al*., 2011, 2014). Thus, more than half of the APase activity in the triple mutant could be attributed to AtPAP10. Although the APase activity in the triple mutant could not completely represent the APase activity of AtPAP10, the results of quantitative analysis of the root-associated APase activity of this triple mutant (Fig. 1C) were consistent with the conclusions obtained from the BCIP staining (Fig. 1A, B). We also analysed the cellular Pi content in the shoots and roots of each sample. The Pi content in R– was only 25% of that in R+, whereas the Pi content in the SR+ was similar to that of R+ (Fig. 1D). The Pi content in SR– was between that in R+ and R–. The Pi content of shoots of the seedlings grown on P+/P– plates (designated split-shoot, or SS) was only slightly lower than that of seedlings grown on P+/P+ plates (designated shoot+ or S+), but was much higher than that of seedlings grown on P–/P– plates (designated shoot– or S–) (Fig. 1D). These results indicated that the induction of the root-associated AtPAP10 activity on SR– was not affected by the high levels of Pi in SR+ and SS. Therefore, we concluded that the induction of AtPAP10 activity on the root surface was independent of Pi status of the whole plant.

### Pi status of the whole plant affects the magnitude but not the triggering of *AtPAP10* transcription in roots

The induction of root-associated AtPAP10 activity by Pi starvation might be controlled at multiple levels, including transcription of *AtPAP10* mRNA, accumulation and secretion of AtPAP10 proteins, and modulation of AtPAP10 enzymatic activity. To determine whether the transcription of the *AtPAP10* gene in roots is affected by Pi status of the whole plant, we quantified the transcripts in the root samples collected from split-root experiments as shown in Fig. 1. The results showed that the mRNA level of *AtPAP10* in SR+ was similar to that in R+ (Fig. 1E); however, the mRNA level of *AtPAP10* was 24% lower in SR– than in R–, indicating that the magnitude of the transcription of the *AtPAP10* gene in roots was affected by the Pi status of the whole plant.

**Fig. 2.**
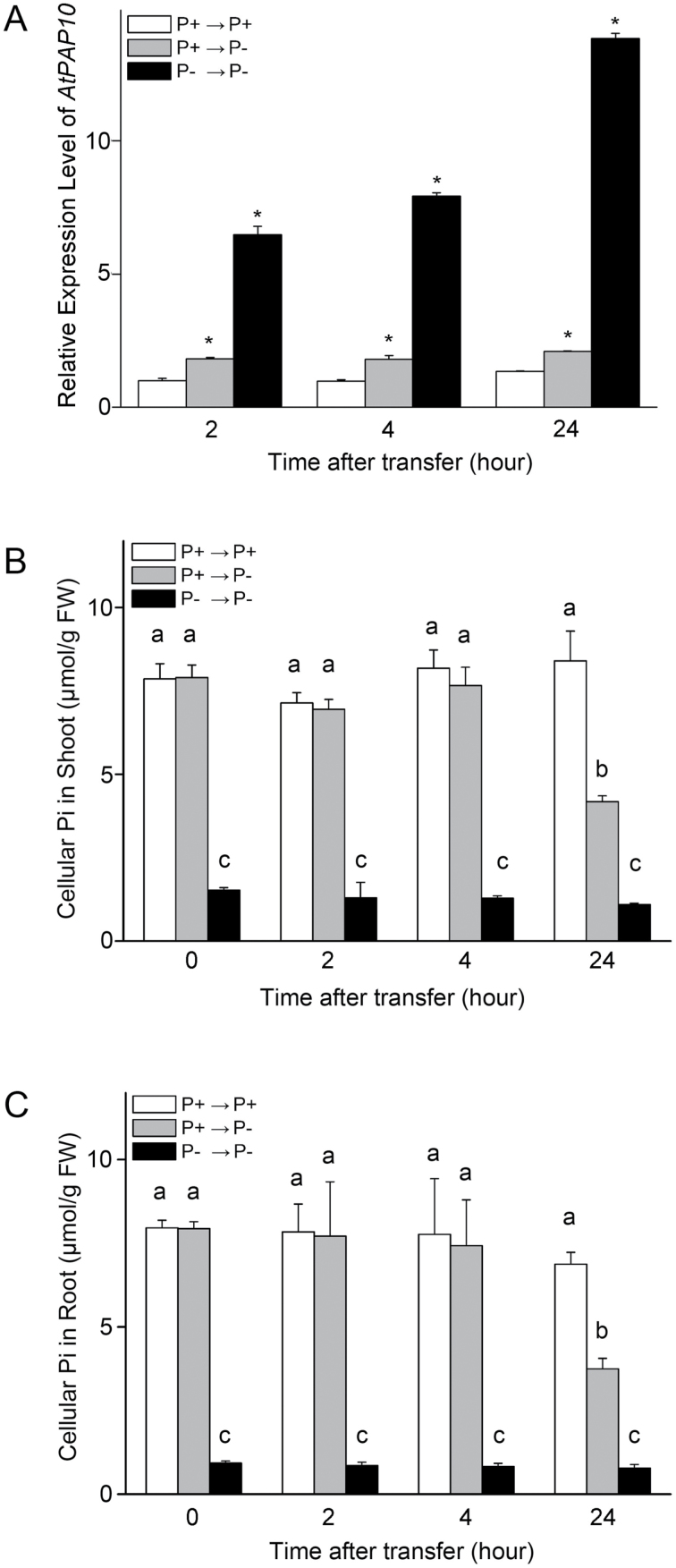
Effects of shoot and root Pi levels on transcription of *AtPAP10* mRNA. Eight-day-old seedlings grown in P+ and P– liquid medium were transferred to P+ or P– medium. Relative expression levels of *AtPAP10* in roots (A) and cellular Pi contents in shoots (B) and roots (C) were analysed at different time intervals after transfer. In A values are the means±SEM of three replicates. The relative expression level of *AtPAP10* at 2h after transfer was set as 1. At each time interval, expression level was compared with that in seedlings transferred from P+ to P+ medium. Asterisks indicate a significant difference from the WT according to a two-sample *t*-test (*P*<0.05). In B and C values are the means±SEM of three replicates. A one-way ANOVA was carried out for the whole data set, and *post hoc* comparisons were conducted using the SPSS Tukey HSD test at *P*<0.05 level.

To further determine whether the triggering of induction of *AtPAP10* transcription in roots is also affected by Pi status of the whole plant, we transferred 8-day-old *Arabidopsis* seedlings grown on P+ agar medium to P– or P+ liquid media and collected samples 2, 4, and 24h after transfer. Quantitative real-time PCR (qPCR) analysis indicated that the induction of *AtPAP10* transcription in roots had already occurred at 2h after transfer (Fig. 2A). However, the decrease of cellular Pi levels in roots and shoots was not observed even at 4h after transfer (Fig. 2B, C). Induction of the other two PSI genes, *At4* and *AtIPS1*, was detected at 4h after transfer, which was also before the decrease of Pi levels in shoots and roots (Supplementary Fig. S3). These results demonstrated that the decrease of Pi levels in shoots and roots was not a prerequisite for the triggering of induction of *AtPAP10* transcription in roots.

Furthermore, at all sampling points, the mRNA levels of *AtPAP10*, *At4*, and *IPS1* in the roots of the seedlings transferred from P– to P– medium were much higher than those of the seedlings transferred from P+ to P– medium (Fig. 2A and Supplementary Fig. S3). This result also indicated that the magnitude of *AtPAP10* transcription in roots was affected by the Pi status of the whole plant.

### Pi status of the whole plant does not affect accumulation of AtPAP10 proteins in roots and change of enzymatic activity of AtPAP10 on the root surface

**Fig. 3.**
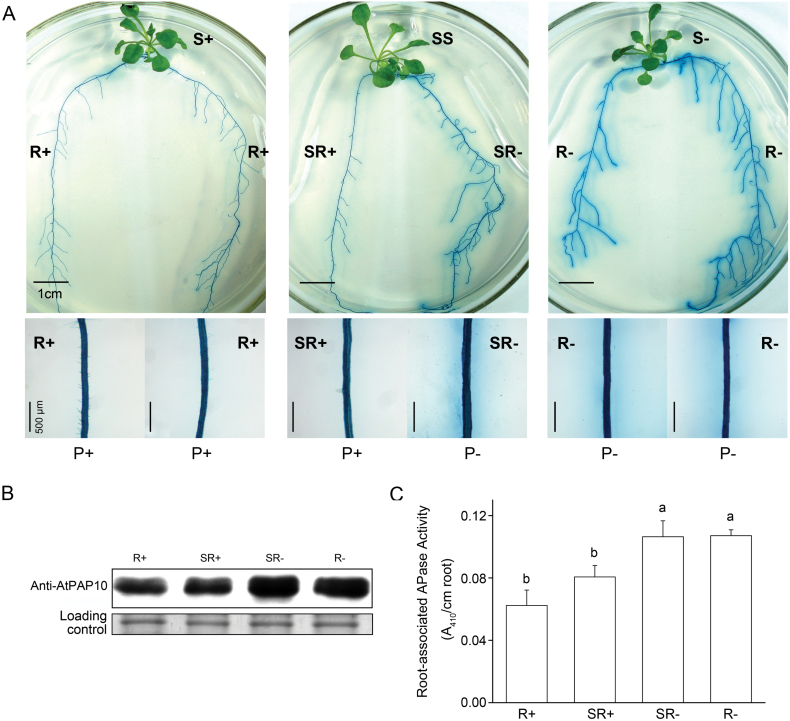
Analysis of AtPAP10 protein levels and root-associated APase activity in split-root experiments. (A) Seventeen-day-old *35S:AtPAP10* seedlings were transferred to compartmentalized dishes with each half of each root system placed in a different compartment. The roots were stained with BCIP to reveal root-associated APase activity 4 d after transfer. Top: Photographs of whole seedlings; Bottom: Close views of the stained roots of the seedlings shown above. (B) Western blot showing protein levels of AtPAP10 in the roots of the *35S::AtPAP10* line grown under different Pi conditions in a split-root experiment. (C) Root-associated APase activity of the *35S:AtPAP10* seedlings in the split-root experiment. Values are the means±SEM of three replicates. A one-way ANOVA was carried out for the whole data set, and *post hoc* comparisons were conducted using the SPSS Tukey HSD test at *P*<0.05 level. In C: R+: Split roots in P+/P+ dishes; R–: Split roots in P–/P– dishes; SR+: Split roots on the P+ side of P+/P– dishes; SR–: Split roots on the P– side of P+/P– dishes.

To investigate whether the accumulation of AtPAP10 protein in roots and the increase of enzymatic activity of AtPAP10 on the root surface are affected by the Pi status of the whole plant, we performed a split-root experiment with a *35S:AtPAP10* transgenic line (the *atpap10* mutant transformed with a *35S:AtPAP10* construct, Wang *et al.*, 2011). This line was used because its transcription of *AtPAP10* mRNA is not responsive to Pi conditions in the environment (Wang *et al.*, 2011). Western blot indicated that the level of AtPAP10 protein was similar in SR– and in R– but was much higher in SR– and R– than in SR+ or R+ (Fig. 3B). This indicated that Pi starvation-enhanced accumulation of AtPAP10 protein was not affected by the Pi status of the whole plant.

The results in Fig. 3 also demonstrated that after AtPAP10 proteins were synthesized in the root cells, the subsequent increase of AtPAP10 enzymatic activity on the root surface was independent of the Pi status of the whole plant. The levels of AtPAP10 proteins in SR– and R– were similar (Fig. 3B), so if the increase in AtPAP10 enzymatic activity were affected by the Pi status of the whole plant, then the AtPAP10 activity on the root surface would be weaker for SR– than for R– owing to a systemic suppression from SR+ or SS. The BCIP staining (Fig. 3A) and quantitative analysis of root-associated APase activity (Fig. 3C), however, indicated that this was not the case. Thus, we concluded that after AtPAP10 proteins were produced in the root cells, the subsequent increase in enzymatic activity of AtPAP10 proteins on the root surface was also unaffected by the Pi status of the whole plant. This increase in AtPAP10 activity on the root surface of *35S:AtPAP10* transgenic line could be due to an increase in the secretion of AtPAP10 proteins, an increase in the enzymatic activity of AtPAP10 proteins, or an increase in both the secretion and enzymatic activity of AtPAP10 proteins.

### Ethylene enhances root-associated AtPAP10 activity mainly by increasing activity of AtPAP10 protein rather than increasing *AtPAP10* transcription and protein accumulation under Pi deficiency

We previously showed that root-associated AtPAP10 activity was enhanced in the *Arabidopsis* mutant, which displays constitutive ethylene responses (*ctr1*, Lei *et al.*, 2011*b*) or overproduces ethylene (*eto1*, Wang *et al.*, 2012), but was reduced in the *Arabidopsis* mutant that is completely insensitive to ethylene (*ein2*, Lei *et al.*, 2011*b*; Wang *et al.*, 2012).

**Fig. 4.**
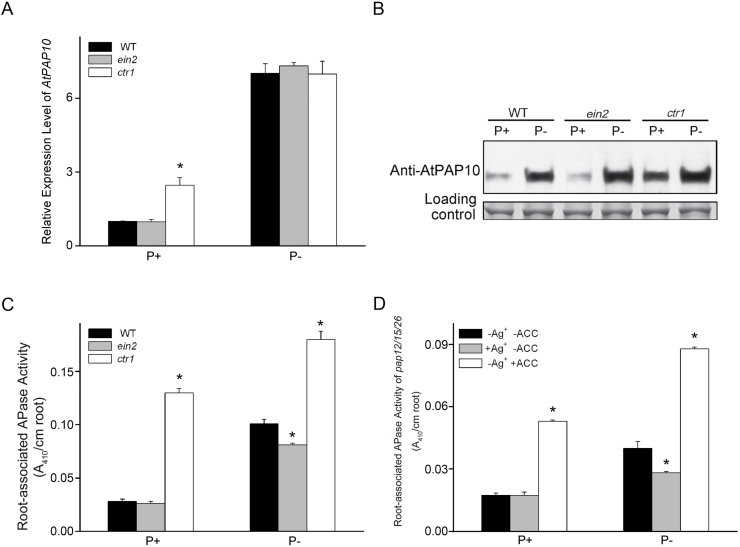
*AtPAP10* mRNA expression levels, AtPAP10 protein levels, and root-associated APase activity in the WT and ethylene-related mutants *ctr1* and *ein2* or in ACC- and Ag^+^-treated WT seedlings under P+ and P– conditions. (A) Quantitative real-time PCR analysis of the expression levels of *AtPAP10* mRNA. (B) Western blot analysis of AtPAP10 proteins. (C) Quantitative analysis of root-associated APase activity. (D) Root-associated APase activity of 7-day-old *atpap10/12/26* seedlings treated or not treated with 1 µM ACC or 5 µM Ag^+^. In A, C, and D values are the means±SEM of three replicates. Asterisks indicate a significant difference from the WT according to a two-sample *t*-test (*P*<0.05).

To determine at which step ethylene is involved in the induction of root-associated AtPAP10 activity, we analysed the effect of ethylene signalling on the transcription of *AtPAP10* mRNA and accumulation of AtPAP10 proteins in the root, as well as the APase activity on the root surface using *ctr1* and *ein2* mutants. On P+ medium, levels of *AtPAP10* mRNA, AtPAP10 protein, and root-associated APase activity were similar between the WT and *ein2* but were higher in *ctr1* (Fig. 4A–C). On P– medium, levels of *AtPAP10* mRNA and AtPAP10 protein did not differ between *ein2* and *ctr1* (Fig. 4A, B); root-associated APase activity, however, was lower in *ein2* and higher in *ctr1* than in the WT (Fig. 4C). Using the *atpap12/atpap15/atpap26* triple mutant, we also tested the effect of ACC (a precursor of ethylene) and Ag^+^ (an inhibitor of ethylene perception) on the induction of APase activity. Quantitative analysis indicated that the induction of root-associated APase activity by Pi starvation was enhanced in the ACC-treated seedlings but reduced in the Ag^+^-treated seedlings (Fig. 4D). These results indicate that under Pi sufficiency, increased ethylene signalling enhances *AtPAP10* transcription, protein accumulation, and enzymatic activity on the root surface. Under Pi deficiency, however, ethylene mainly affects the processes after AtPAP10 protein accumulation, but has no obvious effects on *AtPAP10* transcription and protein accumulation. The regulatory processes after AtPAP10 protein is accumulated might include the secretion of AtPAP10 proteins and enzymatic modification of AtPAP10 proteins on the root surface.

### The effect of ethylene on the induction of root-associated AtPAP10 activity depends on sucrose but the effect of sucrose does not depend on ethylene

**Fig. 5.**
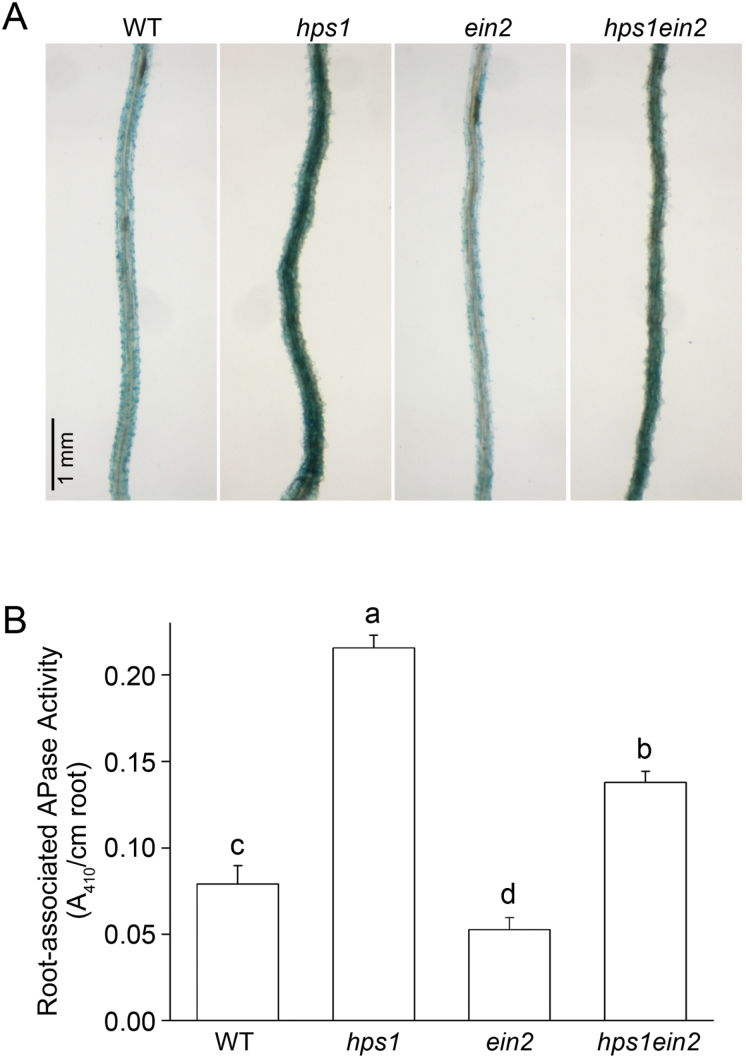
Root-associated APase activity of the WT and various mutants grown under Pi deficiency. (A) Root-associated APase activity of 7-d-old Pi-starved seedlings of WT, and the *hps1*, *ein2,* and *hps1ein2* double mutants as revealed by BCIP staining. (B) Root-associated APase activity in the WT and mutants shown in (A). Values are the means±SEM of three replicates. A one-way ANOVA was carried out for the whole data set, and *post hoc* comparisons were conducted using the SPSS Tukey HSD test at *P*<0.05 level.

Sucrose is a key signal that mediates multiple plant responses to Pi starvation (Liu *et al.*, 2005, 2010; Karthikeyan *et al.*, 2007; Lei *et al.*, 2011*a*). The *Arabidopsis hps1* mutant ectopically overexpresses the *SUCROSE TRANSPORTER 2* (*SUC2*) gene in the root (Lei *et al.*, 2011*a*); therefore, it has a strong capacity to uptake sucrose from the growth medium and accumulates high amounts of sucrose in its root. Under Pi deficiency, the root-associated APase activity is enhanced in *hps1*. In contrast, in the *suc2-5* (Lei *et al.*, 2011*a*) and *pho3* (another allele of the *SUC2* gene, Lloyd and Zakhleniuk, 2004) mutants in which sucrose produced in leaves cannot be transported to roots, the induction of APase activity on the root surface was largely blocked. To determine the relationship between sucrose and ethylene in regulating root-associated APase activity, we constructed the *hps1ein2* double mutant and compared the root-associated APase activity among the WT, *hps1*, *ein2*, and *hps1ein2*. As shown in Figure 5, *hps1* had higher and *ein2* had lower root-associated APase activity than the WT. In *hps1ein*2, the root-associated APase activity was intermediate between that in *hps1* and the WT. Quantitative analysis showed that the levels of sucrose did not differ in *hps1ein2* vs. *hps1* (Supplementary Fig. S4), indicating that the decrease of root-associated APase activity in *hps1ein2* was due to the blockage of ethylene signalling. Taken together, these results suggested two hypotheses: (i) The effect of sucrose on the induction of root-associated APase activity partly depends on ethylene signalling; and (ii) Sucrose and ethylene act in parallel and together have an additive effect.

**Fig. 6.**
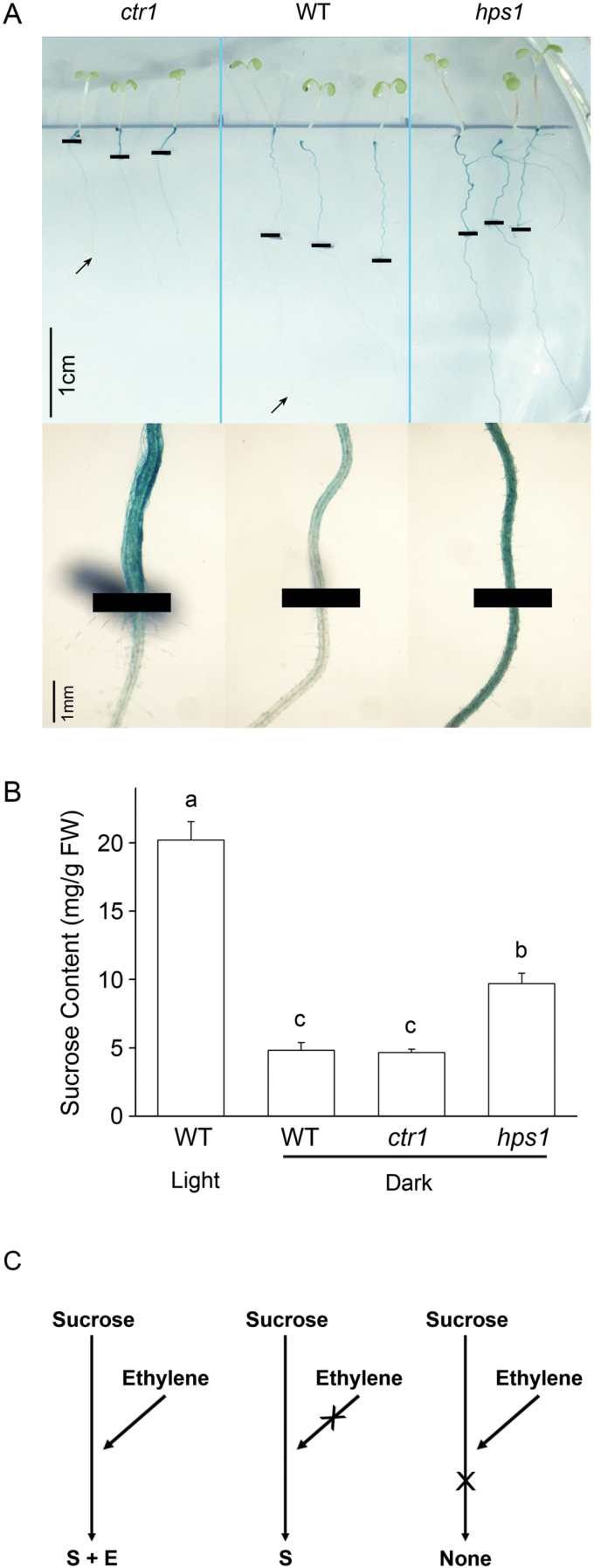
Root-associated APase activity of WT, *ctr1*, and *hps1* in dark treatment experiments. Five-day-old seedlings of the WT, *ctr1*, and *hps1* grown on P+ medium were transferred to P– medium and allowed to grow in the dark. The roots were stained with BCIP 8 d after transfer. (A) Photographs of whole seedlings. Black lines indicate the positions of the root tips when seedlings were transferred to new agar plates, and arrows indicate the new positions of the root tips 8 d after transfer. Bottom: Close views of those parts of the primary roots shown above that grew after transfer. (B) Sucrose content in the roots of seedlings shown above; WT seedlings grown with light were included for comparison. Values are the means±SEM of three replicates. A one-way ANOVA was carried out for the whole data set, and *post hoc* comparisons were conducted using the SPSS Tukey HSD test at *P*<0.05 level. (C) A diagram showing the relationship between sucrose and ethylene. At the bottom of the diagram, “S” means the effect of sucrose and “E” means the effect of ethylene.

To investigate whether the effect of ethylene depends on sucrose, we transferred 5-day-old WT and *ctr1* seedlings grown on P+ medium to P– medium and allowed them to grow in continuous dark for 8 d before BCIP staining. The purpose of this experiment was to determine whether APase activity could still be induced in *ctr1* with a low level of sucrose (sucrose would be consumed after a short period of growth in the dark). Eight days after transfer, blue staining was evident on the surface of the “old roots” (those formed before transfer) but not on the “new roots” (those formed after transfer) of both the WT and *ctr1* (Fig. 6A). (The blue staining on the old roots was probably due to the presence of some sucrose in the old roots before transfer and the APase activity induced on the old roots would then be stabilized as long as the seedlings were grown on P– medium.) The absence of BCIP staining on the “new roots” of both the WT and *ctr1* could be attributed to a low level of sucrose in root cells owing to the long period of growth in the dark or to the lack of an unknown, light-inducible factor. To exclude the second possibility, we performed the same experiment with the *hps1* mutant. When grown in the dark, *hps1* accumulated twice more sucrose in its roots than did the WT or *ctr1* (Fig. 6B). Eight days after transfer, a strong BCIP staining was detected on the surface of the new roots of *hps1* (Fig. 6A). This result demonstrated that the level of sucrose, rather than a putative light-inducible factor, was responsible for the induction of root-associated APase activity. These results also indicated that without sufficient sucrose in the root cells, root-associated APase activity could not be induced even when ethylene signalling was constitutively on. Considering the results obtained with the *hps1ein2* double mutant, we concluded that the effect of ethylene on the induction of root-associated APase activity is completely dependent on sucrose, but that the effect of sucrose on the induction of root-associated APase activity is independent of ethylene. And, when both signalling pathways are turned on, they display an additive effect. A working model for such interaction is shown in Fig. 6C.

**Fig. 7.**
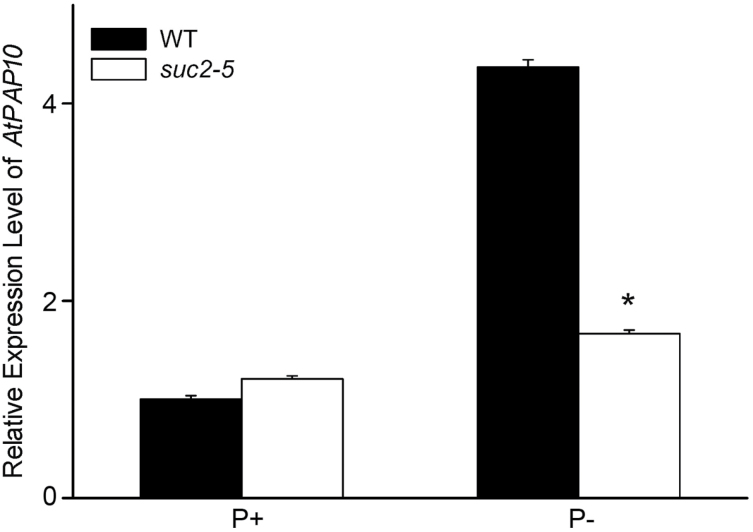
Relative expression levels of *AtPAP10* in the roots of 10-day-old WT and *suc2-5* seedlings under P+ and P– conditions. Values are the means±SEM of three replicates. Asterisks indicate a significant difference according to a two-sample *t*-test (*P*<0.05).

To understand why ethylene could not enhance the APase activity on the root surface in the absence of sucrose, we examined the expression of *AtPAP10* mRNA in the *suc2-5* mutant, which cannot accumulate sucrose in its root tissues when grown on sucrose-free medium (Lei *et al.*, 2011*a*). Our qPCR analysis showed that the induction of *AtPAP10* transcription was largely impaired in *suc2-5* under Pi deficiency (Fig. 7). Because ethylene is involved in the regulatory processes after accumulation of AtPAP10 protein and transcription of *AtPAP10* is dependent on the presence of sucrose, it is reasonable that ethylene cannot enhance the APase activity in the absence of sucrose.

## Discussion

The induction and secretion of APase is a universal adaptive response of plants to Pi starvation (Tran *et al.*, 2010*a*). The goal of this study was to understand the roles of local and systemic signalling in the induction of root-associated AtPAP10 activity under Pi deprivation. Using split-root assays, researchers have found that the PSI genes involved in Pi transport, recycling, and remobilization are systemically regulated (Liu *et al.*, 1998; Burleigh and Harrison, 1999; Hou *et al.*, 2005; Franco-Zorrilla *et al.*, 2005; Liu *et al.*, 2010; Thibaud *et al.*, 2010). Consistent with this view, our split-root assays (Fig. 1E) and seedling transfer experiment (Fig. 2A) showed that a systemic signalling is involved in the control of *AtPAP10* transcription. Before this work, however, it was not known whether a local signalling pathway is also involved in the control of transcription of *AtPAP10* mRNA.

In our split-root experiment, the incomplete systemic suppression of the transcription of *AtPAP10* in SR– (Fig. 1E) suggested that a local signalling pathway might also be involved in the induction of transcription of the *AtPAP10* gene. Additional evidence for this hypothesis was that the induction of *AtPAP10* transcription occurred before the decrease of Pi levels in both shoots and roots (Fig. 2). Hou *et al.* (2005) reported that the induction of *OsIPS1* and *OsIPS2* transcription was evident 4h after whole rice seedlings were transferred from P+ to P– nutrient solution. At that time point, the Pi level in the roots had decreased, whereas the Pi level in the shoots was still unchanged. Based on this result, the authors concluded that a local signalling pathway was involved in the induction of *OsIPS1* and *OsIPS2*, which was triggered by the decrease of root internal Pi. Their conclusion differs from what we observed (Fig. 2). This discrepancy can probably be attributed to their sampling time, which was not short enough after the seedlings had been transferred from the P+ to the P– medium. Based on our results, we propose that both local and systemic signalling pathways are involved in the induction of *AtPAP10* transcription and they may act at the different stages after the plants are exposed to Pi starvation. The local signalling pathway may be initiated at the early stage by a drop in the external concentration of Pi, which is sufficient to trigger the induction of AtPAP10 transcription. Later, a systemic signal may be generated when the Pi levels in the whole plant are reduced, which ultimately determines the magnitude of the induction. Owing to the limited sensitivity of the techniques that are currently available, however, we also cannot exclude the possibility that a slight decrease of the Pi level in the root epidermal cells triggers the induction of *AtPAP10* transcription.

Whereas the transcription of *AtPAP10* mRNA seems to involve a dual signalling pathway, subsequent accumulation of AtPAP10 proteins in the root cells and increase of AtPAP10 activity on the root surface may be solely dependent on a local signalling pathway. Our split-root experiments demonstrated that the accumulation of AtPAP10 protein and change of AtPAP10 enzymatic activity on the root surface are not affected by Pi status of the whole plant (Fig. 3). These results indicated that local and systemic signalling play distinct roles in transcriptional and post-transcriptional regulation of root-associated APase activity under Pi deficiency. In the past, the roles of local and systemic signalling in plant Pi responses have only been analysed for Pi starvation-induced root architecture changes or transcriptional response. This study increases our understanding of the roles of local and systemic signalling in regulating plant responses to Pi starvation.

In our previous work, we showed that ethylene is an important mediator for the induction of AtPAP10 activity under Pi deficiency (Lei *et al.*, 2011*b*; Wang *et al.*, 2012; Yu *et al.*, 2012). Enhanced ethylene signalling does not significantly affect the total intracellular APase activity in root tissues but significantly increases APase activity on the root surface. In the current work, we further showed that under Pi deficiency, ethylene is mainly involved in the control of the secretion of AtPAP10 proteins, the enzymatic activity of AtPAP10 proteins, or both the secretion and enzymatic activity of AtPAP10 proteins. Because these two processes were regulated by local signalling (Fig. 3), ethylene is likely to serve as a local signal acting downstream of external Pi. This conclusion is further supported by a transcriptomic analysis showing that some ethylene biosynthetic and signalling genes are locally induced by Pi starvation (Thibaud *et al.*, 2010).

This work also elucidated how sucrose and ethylene interact to induce the root-associated APase activity under Pi deficiency. In the *suc2-5* mutant, which cannot accumulate sucrose in its roots, the induction of root-associated APase activity is almost completely abolished, whereas in the completely ethylene-insensitive mutant *ein2*, the induction of APase is only partially blocked (Lei *et al*., 2011*a*, 2011*b*; Wang *et al.*, 2012). These results suggest that sucrose and ethylene may have different roles in the induction of root-associated APase activity. Here, we provided the first genetic evidence that the effect of ethylene on the induction of root-associated APase activity is dependent on the presence of sucrose but that the effect of sucrose is not dependent on the presence of ethylene (Fig. 6C). To understand the mechanism behind such interactions, we examined the transcription of *AtPAP10* in the *suc2-5* mutant and found that the induction of *AtPAP10* transcription was almost completely blocked (Fig. 7). Liu *et al.* (2005) also found that in the roots of dark-grown or stem-girdled white lupin and common bean seedlings, the induction of the APase genes *LaSAP1* and *PvHAD1* was largely impaired (Liu *et al.*, 2005, 2010). These results demonstrate that sucrose is essential for the induction of transcription of APase genes under Pi deficiency. Given that ethylene is mainly involved in the processes after AtPAP10 protein accumulation, i.e. protein secretion and change of enzymatic activity on the root surface, it would be expected that the function of sucrose in induction of *AtPAP10* transcription is not dependent on ethylene.

The general criteria used to determine whether a root Pi response is controlled by systemic signalling is to examine whether the level of the response depends on the Pi status of the shoot or whole plant. If a root response occurs before the change of Pi level in the shoot, this response would be regarded as controlled by local signalling. However, with increases in our understanding of Pi sensing and signalling mechanisms, it is now clear that transmission of some systemic signals, such as sucrose and miRNA399, from shoots to roots might occur before the change of Pi levels in shoots. In other words, change of Pi levels in the shoots might not be a prerequisite for the onset of shoot–root communications that systemically control the Pi responses in the roots. Although the classic criteria indicate that the induction of *AtPAP10* transcription is triggered by a decreased level of external Pi through a local signalling pathway, we cannot exclude a role for systemic signalling involved in the triggering of the induction of *AtPAP10* transcription. This is because the induction of *AtPAP10* transcription in roots is largely dependent on the presence of sucrose, a systemic signal translocated from the shoots.

**Fig. 8.**
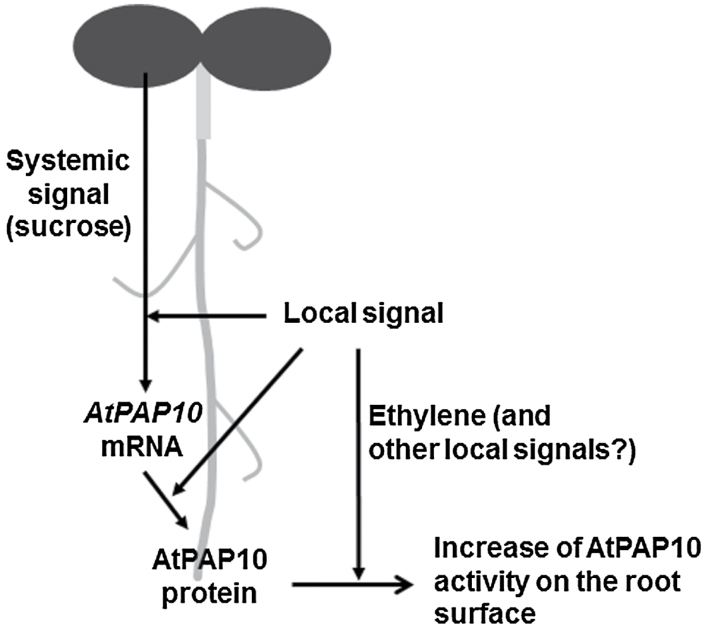
A working model showing the distinct roles of local and systemic signalling in regulating the root-associated APase activity induced by Pi starvation. In this diagram, the increase of AtPAP10 activity on the root surface can be due to an increase in the secretion of AtPAP10 proteins, an increase of enzymatic activity of AtPAP10 proteins, or an increase in both secretion and enzymatic activity of AtPAP10 proteins.

In summary (see the working model in Fig. 8), we have demonstrated that the induction of *AtPAP10* transcription is initiated by a decrease in external Pi levels through a local signalling pathway. The effect of this local signalling pathway is also dependent on the presence of a systemic signal, sucrose, which is translocated from the shoots. Although the Pi status in the whole plant does not affect the triggering of the induction of AtPAP10 transcription, it can affect the magnitude of the induction. Once the AtPAP10 mRNA is synthesized in roots, the subsequent protein accumulation and increase of APase activity on the root surface is solely dependent on the local signalling. Under Pi deficiency, ethylene mainly controls the increase of APase activity of AtPAP10 proteins rather than *AtPAP10* transcription and protein accumulation, and is likely to serve as a local signal. Furthermore, we showed that ethylene’s effect is dependent on sucrose but the effect of sucrose is not dependent on ethylene. Thus, our study has revealed that both local and systemic signalling pathways are involved in the regulation of root-associated APase activity under Pi deficiency and that these two pathways play distinct roles at different regulatory steps. Although these results provide insight into how local and systemic signalling regulate plant responses to Pi starvation at multiple levels, additional research is needed to identify the molecular nature of both local and systemic signalling pathways at different regulatory steps.

## Supplementary data

Supplementary data are available at *JXB* online

Figure S1. Western-blot analysis of AtPAP10 protein in WT, *atpap10* mutants and the *35S::AtPAP10* line.

Figure S2. Root-associated APase activity of the WT and *atpap12/15/26* triple mutant under P+ and P– conditions.

Figure S3. Induction of *At4* and *IPS1* gene expression in the transfer experiments.

Figure S4. Sucrose contents of WT, *hps1*, *ein2* and *hps1ein2* double mutants.

Table S1. Sequences of the primers used for quantitative Real-time PCR.

Supplementary Data

## Abbreviations:

ACC: 1-aminocyclopropane-1-carboxylic acid
APase: acid phosphatase
AtPAPs: *Arabidopsis* purple phosphatases
BCIP: 5-bromo-4- chloro-3-indolyl-phosphate
ctr1: *constitutive triple responses 1*
ein2: *ethylene insensitive 2*
eto1: *ethylene overproduction 1*
P: phosphorus
PAPs: purple acid phosphatases
Pi: phosphate
*p*NPP: *p*-nitrophenol phosphate
PSI: phosphate starvation-induced
qPCR: quantitative real-time PCR
WT: wild type.
