# Supplementary material for: A major root-associated acid phosphatase in Arabidopsis, AtPAP10, is regulated by both local and systemic signals under phosphate starvation
Source: J Exp Bot. 2014 Sep 20;65(22):6577–88. doi: 10.1093/jxb/eru377 (PMC4246188; doi:10.1093/jxb/eru377)
Supplement: Supplementary Data [file supp_65_22_6577__index.html]

A major root-associated acid phosphatase in Arabidopsis, AtPAP10, is regulated by both local and systemic signals under phosphate starvation — A major root-associated acid phosphatase in Arabidopsis, AtPAP10, is regulated by both local and systemic signals under phosphate starvation — Supplementary Data 

# A major root-associated acid phosphatase in *Arabidopsis*, AtPAP10, is regulated by both local and systemic signals under phosphate starvation

## Supplementary Data

Data files

**Files in this Data Supplement:**

- Supplementary Data - Supplementary Data
