# Supplementary material for: A major root-associated acid phosphatase in Arabidopsis, AtPAP10, is regulated by both local and systemic signals under phosphate starvation
Source: J Exp Bot. 2014 Sep 20;65(22):6577–88. doi: 10.1093/jxb/eru377 (PMC4246188; doi:10.1093/jxb/eru377)
Supplement: Supplementary Data [file supp_eru377_jexbot129965_file001.pdf]

Zhang et al. Supplemental figure S1

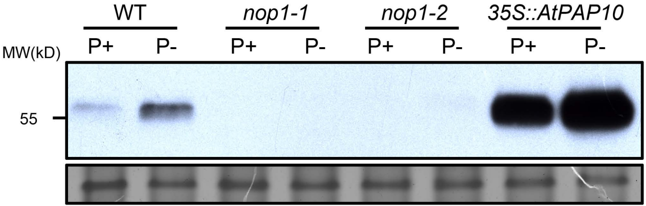

Supplemental Figure S1. Western blot analysis of AtPAP10 protein levels in the 9-day-old seedlings of the WT, the *atpap10* mutants *nop1-1* and *nop1-2*, and the *35S::AtPAP10* line using anti-AtPAP10 antibodies.

Zhang et al. Supplemental figure S2

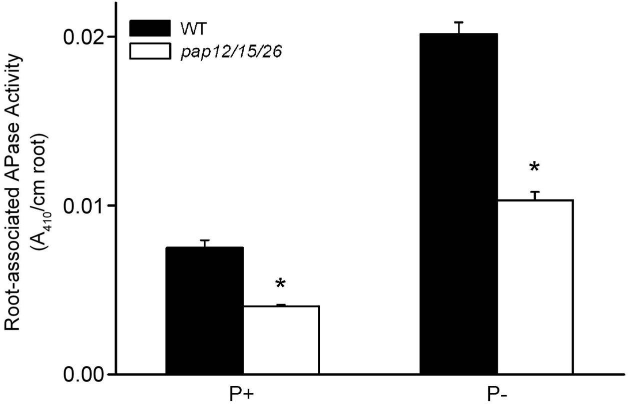

Supplemental Figure S2. Root-associated APase activity of 9-day-old seedlings of the WT and *atpap12/15/26* triple mutant under P+ and P- conditions. Values are the means ± SE of three replicates. Asterisks indicate a significant difference according to a two-sample *t*-test (P<0.05).

Zhang et al. Supplemental figure S3

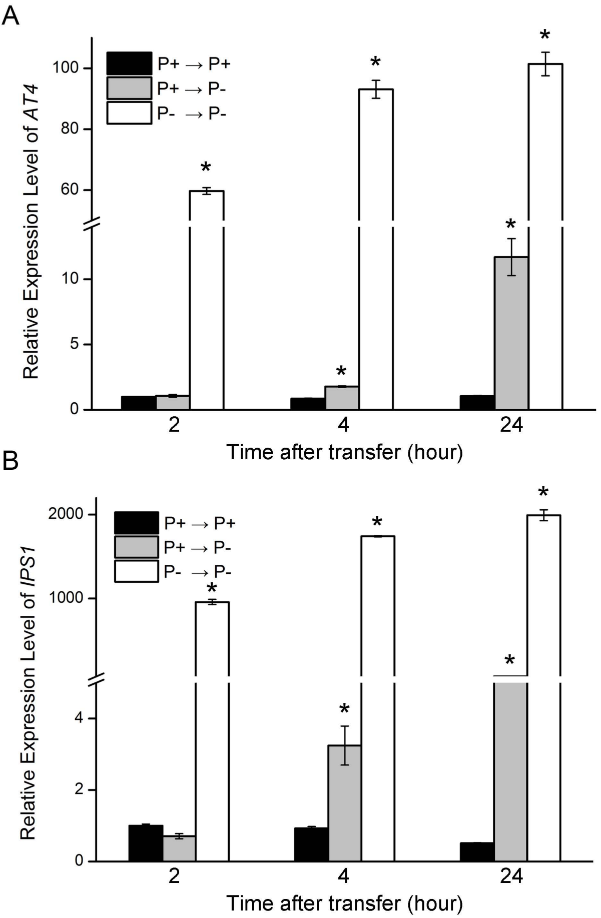

Supplemental Figure S3. Induction of *At4* and *IPS1* gene expression in the transfer experiments. Eight-day-old seedlings grown in P+ and P- medium were transferred to P+ or P- liquid medium. The relative expression levels of *At4* (A) and *IPS1* (B) were analyzed by quantitative real-time PCR at different time intervals after transfer. Values are the means ± SE of three replicates. The relative expression level of *At4* and *IPS1* at 2 h after transfer was set as 1. At each time interval, expression was compared to the expression in seedlings transferred from P+ to P+ medium. Asterisks indicate a significant difference according to a two-sample *t*-test ($P<0.05$).

Zhang et al. Supplemental figure S4

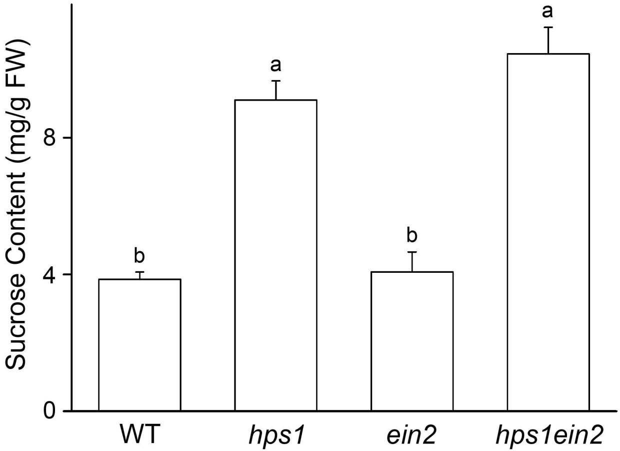

Supplemental Figure S4. Sucrose contents of WT and various mutant seedlings grown under Pi deficiency as described in Figure 7.

Supplementary Table S1. Sequences of the primers used for quantitative Real-time PCR.

Gene	Forward primer	Reverse Primer
AtPAP10	5'-TCCTGTTGATGATTCTCCTTCTTG-3'	5'-ATTCATTTATTTGGATGGTTGTTCA-3'
AT4	5'-TGGCCCCAAACACAAGAG -3'	5'-CGAACATTCACAATCATAATCTCC -3'
IPS1	5'-AGACTGCAGAAGGCTGATTCAGA-3'	5'-TTGCCCAATTTCTAGAGGGAGA -3'
Actin	5'-GACCTTGCTGGACGTGACCTTAC-3'	5'-TAGTCAACAGCAACAAAGGAGAGC-3'
